# *N*-(3-Meth­oxy­benzo­yl)-2-methyl­benzene­sulfonamide

**DOI:** 10.1107/S1600536813018539

**Published:** 2013-07-10

**Authors:** S. Sreenivasa, D Darshan, M. Prakash Shet, N. R. Mohan, Vijith Kumar, P. A. Suchetan

**Affiliations:** a Department of Studies and Research in Chemistry, Tumkur University, Tumkur, Karnataka 572 103, India; b University College of Science, Tumkur University, Tumkur, India; c Department of Studies and Research in Chemistry, U.C.S., Tumkur University, Tumkur, Karnataka 572 103, India; d Solid State and Structural Chemistry Unit, Indian Institute of Science, Bangalore, India

## Abstract

In the title compound, C_15_H_15_NO_4_S, the dihedral angle between the methyl- and meth­oxy-substituted benzene rings is 88.99 (12)°. An intra­molecular C—H⋯O hydrogen bond occurs. In the crystal, adjacent mol­ecules form inversion-related dimers through strong N—H⋯O hydrogen bonds, generating *R*_2_^2^(8) loops. The dimers are further connected through C—H⋯O inter­actions that form *C*(8) chains parallel to (001). Mol­ecules are also connected through other C—H⋯O hydrogen bonds along the *b* axis, forming additional *C*(8) chains. Two aromatic π–π stacking inter­actions [centroid–centroid separations = 3.6150 (1) and 3.6837 (1) Å] generate a three-dimensional architecture.

## Related literature

For similar structures, see: Gowda *et al.* (2010 ▶); Suchetan *et al.* (2010 ▶, 2011 ▶).
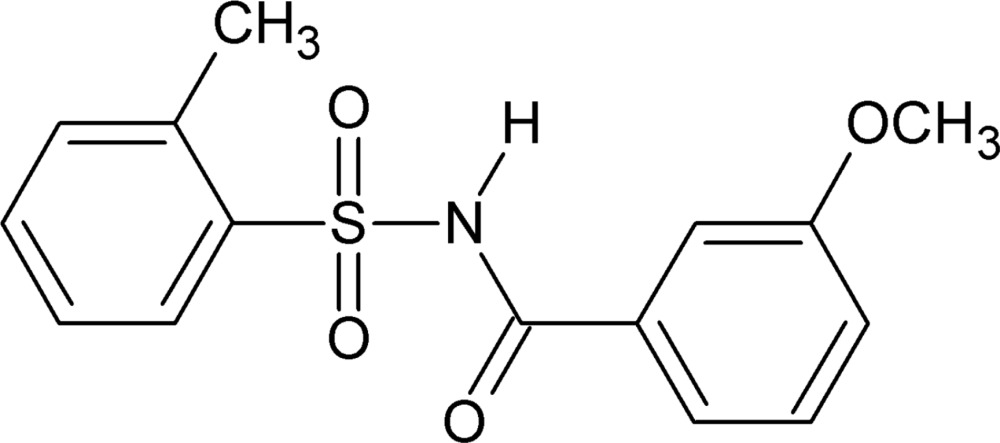


## Experimental

### 

#### Crystal data

C_15_H_15_NO_4_S
*M* *_r_* = 305.34
Monoclinic, 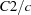

*a* = 26.713 (5) Å
*b* = 7.3717 (4) Å
*c* = 19.636 (3) Å
β = 131.21 (3)°
*V* = 2908.7 (7) Å^3^
*Z* = 8
Mo *K*α radiation
μ = 0.24 mm^−1^
*T* = 293 K
0.33 × 0.27 × 0.22 mm

#### Data collection

Bruker APEXII CCD area-detector diffractometer
5294 measured reflections
2558 independent reflections
1970 reflections with *I* > 2σ(*I*)
*R*_int_ = 0.027

#### Refinement

*R*[*F* ^2^ > 2σ(*F* ^2^)] = 0.045
*wR*(*F* ^2^) = 0.118
*S* = 1.04
2558 reflections
196 parameters
H atoms treated by a mixture of independent and constrained refinement
Δρ_max_ = 0.19 e Å^−3^
Δρ_min_ = −0.35 e Å^−3^

### 

Data collection: *APEX2* (Bruker, 2009 ▶); cell refinement: *APEX2* and *SAINT-Plus* (Bruker, 2009 ▶); data reduction: *SAINT-Plus* and *XPREP* (Bruker, 2009 ▶); program(s) used to solve structure: *SHELXS97* (Sheldrick, 2008 ▶); program(s) used to refine structure: *SHELXL97* (Sheldrick, 2008 ▶); molecular graphics: *Mercury* (Macrae *et al.*, 2008 ▶); software used to prepare material for publication: *SHELXL97*.

## Supplementary Material

Crystal structure: contains datablock(s) I, New_Global_Publ_Block. DOI: 10.1107/S1600536813018539/sj5340sup1.cif

Structure factors: contains datablock(s) I. DOI: 10.1107/S1600536813018539/sj5340Isup2.hkl

Supplementary material file. DOI: 10.1107/S1600536813018539/sj5340Isup3.cml

Additional supplementary materials:  crystallographic information; 3D view; checkCIF report

## Figures and Tables

**Table 1:** Hydrogen-bond geometry (Å, °)

*D*—H⋯*A*	*D*—H	H⋯*A*	*D*⋯*A*	*D*—H⋯*A*
N1—H*N*1⋯O2^i^	0.80 (3)	2.15 (4)	2.929 (4)	166
C3—H3⋯O3^ii^	0.93	2.60	3.463 (3)	155
C10—H10⋯O2^i^	0.93	2.60	3.404 (3)	145
C15—H15*B*⋯O3^iii^	0.96	2.36	3.323 (3)	178
C6—H6⋯O1	0.93	2.46	2.861 (4)	106

Symmetry codes: (i) 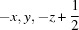; (ii) 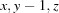; (iii) 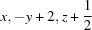.

**Fig. 1.**
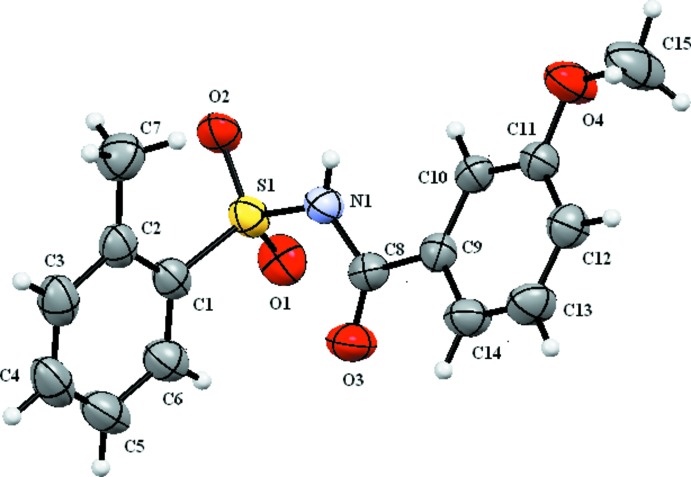
Molecular structure of the title compound, showing the atom-labeling scheme. Displacement ellipsoids are drawn at the 50% probability level.

**Fig. 2.**
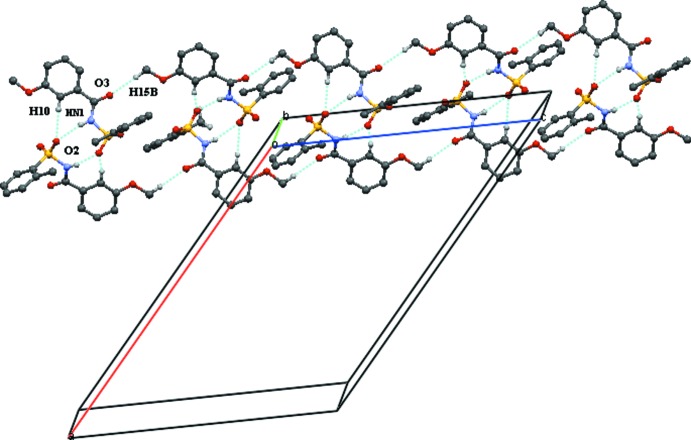
Molecular packing of (I) with hydrogen bonding shown as dashed lines. Hydrogen atoms bound to carbon are omitted for clarity.

**Fig. 3.**
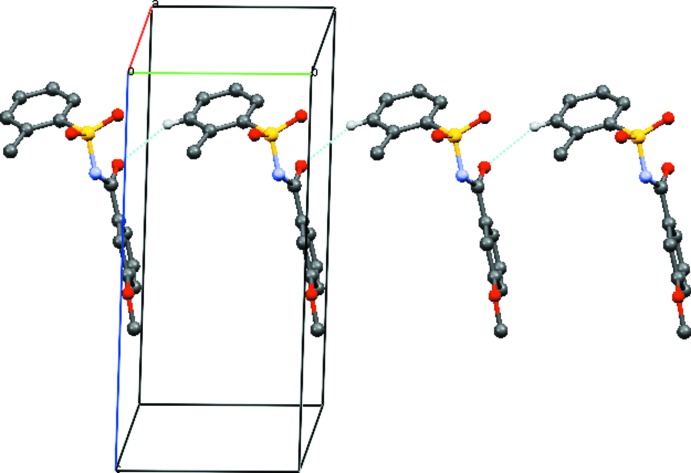
Display of C—H···O interactions among molecules along b axis forming C(8) chains.

**Fig. 4.**
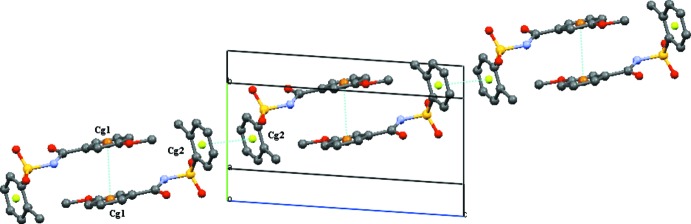
Stacking of molecules through Cg···Cg interactions. Cg1 and Cg2 are the centroids of the carbonyl and sulfonyl bounded benzene rings respectively.

## supplementary crystallographic information

### Comment

As a part of our continued efforts to study the crystal structures of N-(aroyl)-arylsulfonamides (Suchetan *et al.*, 2010, 2011), we report herein the crystal structure of the title compound (I).

In the title compound, C_15_H_15_NO_4_S, the dihedral angle between the benzene rings is 88.99°. In the molecule, the conformation between the N-H bond and the ortho-methyl group in the sulfonyl benzene ring is *syn*. This is similar to what is observed in N-(benzoyl)-2-methylbenzenesulfonamide (II, Suchetan *et al.*, 2010), N-(3-chlorobenzoyl)- 2-methylbenzenesulfonamide (III, Suchetan *et al.*, 2011) and N-(3-methylbenzoyl)-2-methylbenzenesulfonamide (IV, Gowda *et al.*, 2010). Similarly, the conformation between the N-H bond and the meta-methoxy group in the benzoyl ring is *syn*, also similar to the conformation in III (Suchetan *et al.*, 2011) and IV (Gowda *et al.*, 2010).

Adjacent molecules form inversion related dimers through strong N1–HN1···O2 hydrogen bonds, Table 1, generating R^2^_2_(8) loops. The dimers are further connected through intermolecular C10—H10···O2 interactions that form C(8) chains parallel to (001). Molecules are also connected through other intermolecular C15—H15B···O3 hydrogen bonds along the *b* axis forming additional C(8) chains. Two aromatic π–π stacking interactions (centroid- centroid separations 3.6150 (1) Å and 3.6837 (1) Å) are also observed.

### Experimental

The title compound was prepared by refluxing a mixture of 3-methoxybenzoic acid, 2-methylbenzenesulfonamide and phosphorus oxychloride, POCl_3_, for 2 h on a water bath. The resultant mixture was cooled and poured into ice cold water. The solid obtained was filtered and washed thoroughly with water and then dissolved in sodium bicarbonate solution. The compound was later reprecipitated by acidifying the filtered solution with dilute HCl. The filtered and dried solid was recrystallized to the constant melting point (423 K).

Colorless prisms of (I) were obtained from a slow evaporation of its ethanolic solution at room temperature.

### Refinement

The H atom of the NH group was located in a difference map and later refined freely. The other H atoms were positioned with idealized geometry using a riding model with C—H = 0.93 Å. All H atoms were refined with isotropic displacement parameters (set to 1.2 times of the *U*_eq_ of the parent atom).

### Crystal data

**Table:**

C_15_H_15_NO_4_S	Prism
*M**_r_* = 305.34	*D*_x_ = 1.394 Mg m^−^^3^
Monoclinic, *C*2/*c*	Melting point: 423 K
Hall symbol: -C 2yc	Mo *K*α radiation, λ = 0.71073 Å
*a* = 26.713 (5) Å	Cell parameters from 1232 reflections
*b* = 7.3717 (4) Å	θ = 2.8–25.0°
*c* = 19.636 (3) Å	µ = 0.24 mm^−^^1^
β = 131.21 (3)°	*T* = 293 K
*V* = 2908.7 (7) Å^3^	Prism, colourless
*Z* = 8	0.33 × 0.27 × 0.22 mm
*F*(000) = 1280	

### Data collection

**Table:**

Bruker APEXII CCD area-detector diffractometer	1970 reflections with *I* > 2σ(*I*)
Radiation source: fine-focus sealed tube	*R*_int_ = 0.027
Graphite monochromator	θ_max_ = 25.0°, θ_min_ = 2.8°
φ and ω scans	*h* = −31→31
5294 measured reflections	*k* = −8→8
2558 independent reflections	*l* = −14→23

### Refinement

**Table:**

Refinement on *F*^2^	Primary atom site location: structure-invariant direct methods
Least-squares matrix: full	Secondary atom site location: difference Fourier map
*R*[*F*^2^ > 2σ(*F*^2^)] = 0.045	Hydrogen site location: inferred from neighbouring sites
*wR*(*F*^2^) = 0.118	H atoms treated by a mixture of independent and constrained refinement
*S* = 1.04	*w* = 1/[σ^2^(*F*_o_^2^) + (0.0544*P*)^2^ + 0.0171*P*] where *P* = (*F*_o_^2^ + 2*F*_c_^2^)/3
2558 reflections	(Δ/σ)_max_ = 0.001
196 parameters	Δρ_max_ = 0.19 e Å^−^^3^
0 restraints	Δρ_min_ = −0.35 e Å^−^^3^
0 constraints	

### Special details

**Table:**

Geometry. All s.u.'s (except the s.u. in the dihedral angle between two l.s. planes) are estimated using the full covariance matrix. The cell s.u.'s are taken into account individually in the estimation of s.u.'s in distances, angles and torsion angles; correlations between s.u.'s in cell parameters are only used when they are defined by crystal symmetry. An approximate (isotropic) treatment of cell s.u.'s is used for estimating s.u.'s involving l.s. planes.
Refinement. Refinement of *F*^2^ against ALL reflections. The weighted *R*-factor *wR* and goodness of fit *S* are based on *F*^2^, conventional *R*-factors *R* are based on *F*, with *F* set to zero for negative *F*^2^. The threshold expression of *F*^2^ > 2σ(*F*^2^) is used only for calculating *R*-factors(gt) *etc*. and is not relevant to the choice of reflections for refinement. *R*-factors based on *F*^2^ are statistically about twice as large as those based on *F*, and *R*- factors based on ALL data will be even larger.

### Fractional atomic coordinates and isotropic or equivalent isotropic displacement parameters (Å^2^)

**Table:**

	*x*	*y*	*z*	*U*_iso_*/*U*_eq_	
HN1	0.0737 (12)	0.796 (3)	0.2976 (17)	0.055 (8)*	
C1	0.05769 (11)	0.6312 (3)	0.13024 (14)	0.0444 (5)	
C9	0.18751 (12)	0.9525 (3)	0.40254 (15)	0.0425 (5)	
C10	0.16739 (12)	0.9650 (3)	0.45194 (16)	0.0479 (6)	
H10	0.1231	0.9451	0.4237	0.057*	
C8	0.14165 (12)	0.9088 (3)	0.30428 (16)	0.0467 (6)	
C2	0.06638 (11)	0.4482 (3)	0.15570 (16)	0.0488 (6)	
C6	0.07526 (12)	0.6977 (3)	0.08233 (16)	0.0546 (6)	
H6	0.0689	0.8195	0.0662	0.066*	
C14	0.25399 (13)	0.9826 (3)	0.44521 (17)	0.0529 (6)	
H14	0.2678	0.9748	0.4125	0.063*	
C11	0.21314 (12)	1.0071 (3)	0.54308 (17)	0.0496 (6)	
C13	0.29856 (13)	1.0235 (3)	0.53520 (18)	0.0602 (7)	
H13	0.3429	1.0430	0.5635	0.072*	
C12	0.27921 (12)	1.0365 (3)	0.58542 (17)	0.0529 (6)	
H12	0.3102	1.0646	0.6468	0.063*	
C5	0.10253 (13)	0.5803 (4)	0.05870 (18)	0.0653 (7)	
H5	0.1146	0.6233	0.0267	0.078*	
C4	0.11146 (14)	0.4011 (4)	0.08281 (18)	0.0666 (8)	
H4	0.1296	0.3227	0.0670	0.080*	
C3	0.09384 (13)	0.3358 (3)	0.13017 (18)	0.0601 (7)	
H3	0.1004	0.2136	0.1457	0.072*	
C7	0.04728 (14)	0.3666 (3)	0.20628 (19)	0.0651 (7)	
H7A	0.0607	0.4466	0.2543	0.098*	
H7B	0.0691	0.2516	0.2312	0.098*	
H7C	−0.0001	0.3496	0.1655	0.098*	
C15	0.23539 (16)	1.0486 (5)	0.68140 (19)	0.0927 (11)	
H15A	0.2674	0.9521	0.7117	0.139*	
H15B	0.2121	1.0546	0.7032	0.139*	
H15C	0.2578	1.1616	0.6934	0.139*	
O1	0.00755 (9)	0.9515 (2)	0.10733 (11)	0.0614 (5)	
O2	−0.02956 (7)	0.7037 (2)	0.14851 (11)	0.0527 (4)	
O3	0.15524 (9)	0.9414 (2)	0.25752 (12)	0.0646 (5)	
O4	0.18890 (10)	1.0151 (3)	0.58597 (13)	0.0771 (6)	
S1	0.02251 (3)	0.78797 (8)	0.15606 (4)	0.0461 (2)	
N1	0.08109 (10)	0.8300 (3)	0.26647 (13)	0.0456 (5)	

### Atomic displacement parameters (Å^2^)

**Table:**

	*U*^11^	*U*^22^	*U*^33^	*U*^12^	*U*^13^	*U*^23^
C1	0.0325 (12)	0.0577 (14)	0.0369 (12)	−0.0007 (11)	0.0202 (11)	−0.0079 (10)
C9	0.0428 (13)	0.0396 (11)	0.0460 (14)	−0.0036 (10)	0.0296 (12)	−0.0046 (10)
C10	0.0408 (14)	0.0560 (13)	0.0514 (15)	−0.0117 (11)	0.0323 (13)	−0.0128 (11)
C8	0.0477 (15)	0.0498 (13)	0.0500 (15)	−0.0079 (11)	0.0354 (14)	−0.0059 (11)
C2	0.0370 (13)	0.0540 (13)	0.0470 (14)	−0.0035 (11)	0.0240 (13)	−0.0114 (11)
C6	0.0477 (15)	0.0703 (16)	0.0468 (15)	0.0002 (12)	0.0315 (14)	−0.0041 (12)
C14	0.0455 (15)	0.0674 (15)	0.0538 (16)	−0.0046 (12)	0.0361 (14)	−0.0031 (12)
C11	0.0479 (15)	0.0570 (13)	0.0509 (15)	−0.0084 (12)	0.0355 (14)	−0.0110 (11)
C13	0.0386 (14)	0.0809 (17)	0.0573 (17)	−0.0073 (13)	0.0299 (14)	−0.0053 (13)
C12	0.0424 (15)	0.0643 (15)	0.0435 (15)	−0.0053 (12)	0.0247 (13)	−0.0060 (11)
C5	0.0528 (17)	0.100 (2)	0.0516 (16)	−0.0027 (16)	0.0381 (15)	−0.0121 (15)
C4	0.0513 (17)	0.087 (2)	0.0610 (18)	0.0025 (15)	0.0369 (16)	−0.0217 (15)
C3	0.0491 (16)	0.0606 (15)	0.0614 (17)	0.0022 (13)	0.0324 (15)	−0.0135 (13)
C7	0.0658 (19)	0.0565 (15)	0.075 (2)	−0.0007 (13)	0.0473 (18)	0.0007 (13)
C15	0.071 (2)	0.164 (3)	0.0525 (19)	−0.016 (2)	0.0442 (19)	−0.0258 (19)
O1	0.0668 (12)	0.0571 (9)	0.0569 (12)	0.0121 (9)	0.0393 (11)	0.0084 (8)
O2	0.0360 (9)	0.0696 (10)	0.0542 (10)	−0.0035 (8)	0.0305 (9)	−0.0118 (8)
O3	0.0606 (12)	0.0922 (13)	0.0561 (12)	−0.0179 (10)	0.0449 (11)	−0.0102 (9)
O4	0.0531 (12)	0.1345 (16)	0.0517 (12)	−0.0218 (11)	0.0380 (11)	−0.0281 (11)
S1	0.0400 (4)	0.0541 (4)	0.0427 (4)	0.0021 (3)	0.0267 (3)	−0.0040 (3)
N1	0.0450 (12)	0.0577 (12)	0.0394 (12)	−0.0091 (9)	0.0301 (11)	−0.0074 (9)

### Geometric parameters (Å, º)

**Table:**

C1—C6	1.388 (3)	C13—H13	0.9300
C1—C2	1.404 (3)	C12—H12	0.9300
C1—S1	1.762 (2)	C5—C4	1.370 (4)
C9—C10	1.388 (3)	C5—H5	0.9300
C9—C14	1.394 (3)	C4—C3	1.376 (4)
C9—C8	1.487 (3)	C4—H4	0.9300
C10—C11	1.382 (3)	C3—H3	0.9300
C10—H10	0.9300	C7—H7A	0.9600
C8—O3	1.213 (3)	C7—H7B	0.9600
C8—N1	1.387 (3)	C7—H7C	0.9600
C2—C3	1.400 (3)	C15—O4	1.431 (3)
C2—C7	1.509 (3)	C15—H15A	0.9600
C6—C5	1.393 (3)	C15—H15B	0.9600
C6—H6	0.9300	C15—H15C	0.9600
C14—C13	1.363 (3)	O1—S1	1.4232 (16)
C14—H14	0.9300	O2—S1	1.4391 (16)
C11—O4	1.360 (3)	S1—N1	1.666 (2)
C11—C12	1.385 (3)	N1—HN1	0.79 (2)
C13—C12	1.386 (3)		
			
C6—C1—C2	122.0 (2)	C4—C5—H5	120.2
C6—C1—S1	116.49 (18)	C6—C5—H5	120.2
C2—C1—S1	121.48 (17)	C5—C4—C3	120.7 (2)
C10—C9—C14	119.7 (2)	C5—C4—H4	119.6
C10—C9—C8	123.6 (2)	C3—C4—H4	119.6
C14—C9—C8	116.6 (2)	C4—C3—C2	121.9 (2)
C11—C10—C9	120.0 (2)	C4—C3—H3	119.1
C11—C10—H10	120.0	C2—C3—H3	119.1
C9—C10—H10	120.0	C2—C7—H7A	109.5
O3—C8—N1	120.2 (2)	C2—C7—H7B	109.5
O3—C8—C9	122.5 (2)	H7A—C7—H7B	109.5
N1—C8—C9	117.3 (2)	C2—C7—H7C	109.5
C3—C2—C1	116.3 (2)	H7A—C7—H7C	109.5
C3—C2—C7	119.0 (2)	H7B—C7—H7C	109.5
C1—C2—C7	124.7 (2)	O4—C15—H15A	109.5
C1—C6—C5	119.4 (2)	O4—C15—H15B	109.5
C1—C6—H6	120.3	H15A—C15—H15B	109.5
C5—C6—H6	120.3	O4—C15—H15C	109.5
C13—C14—C9	119.5 (2)	H15A—C15—H15C	109.5
C13—C14—H14	120.2	H15B—C15—H15C	109.5
C9—C14—H14	120.2	C11—O4—C15	117.5 (2)
O4—C11—C10	115.8 (2)	O1—S1—O2	118.18 (11)
O4—C11—C12	124.0 (2)	O1—S1—N1	108.93 (10)
C10—C11—C12	120.2 (2)	O2—S1—N1	103.29 (10)
C14—C13—C12	121.4 (2)	O1—S1—C1	109.21 (11)
C14—C13—H13	119.3	O2—S1—C1	110.47 (10)
C12—C13—H13	119.3	N1—S1—C1	105.95 (11)
C11—C12—C13	119.1 (2)	C8—N1—S1	122.61 (17)
C11—C12—H12	120.4	C8—N1—HN1	120.7 (19)
C13—C12—H12	120.4	S1—N1—HN1	116.5 (19)
C4—C5—C6	119.6 (2)		
			
C14—C9—C10—C11	0.0 (3)	C5—C4—C3—C2	−0.1 (4)
C8—C9—C10—C11	−179.7 (2)	C1—C2—C3—C4	0.1 (4)
C10—C9—C8—O3	161.3 (2)	C7—C2—C3—C4	179.1 (2)
C14—C9—C8—O3	−18.4 (3)	C10—C11—O4—C15	176.7 (2)
C10—C9—C8—N1	−17.9 (3)	C12—C11—O4—C15	−2.7 (4)
C14—C9—C8—N1	162.4 (2)	O2—O2—S1—O1	0.00 (5)
C6—C1—C2—C3	0.0 (3)	O2—O2—S1—N1	0.00 (6)
S1—C1—C2—C3	178.99 (18)	O2—O2—S1—C1	0.00 (4)
C6—C1—C2—C7	−178.9 (2)	C6—C1—S1—O1	10.5 (2)
S1—C1—C2—C7	0.1 (3)	C2—C1—S1—O1	−168.48 (18)
C2—C1—C6—C5	−0.2 (4)	C6—C1—S1—O2	142.13 (18)
S1—C1—C6—C5	−179.17 (18)	C2—C1—S1—O2	−36.9 (2)
C10—C9—C14—C13	0.2 (3)	C6—C1—S1—O2	142.13 (18)
C8—C9—C14—C13	179.9 (2)	C2—C1—S1—O2	−36.9 (2)
C9—C10—C11—O4	−179.6 (2)	C6—C1—S1—N1	−106.65 (19)
C9—C10—C11—C12	−0.1 (3)	C2—C1—S1—N1	74.3 (2)
C9—C14—C13—C12	−0.2 (4)	O3—C8—N1—S1	−3.9 (3)
O4—C11—C12—C13	179.5 (2)	C9—C8—N1—S1	175.28 (15)
C10—C11—C12—C13	0.1 (4)	O1—S1—N1—C8	−54.9 (2)
C14—C13—C12—C11	0.1 (4)	O2—S1—N1—C8	178.66 (18)
C1—C6—C5—C4	0.2 (4)	O2—S1—N1—C8	178.66 (18)
C6—C5—C4—C3	0.0 (4)	C1—S1—N1—C8	62.5 (2)

### Hydrogen-bond geometry (Å, º)

**Table:**

*D*—H···*A*	*D*—H	H···*A*	*D*···*A*	*D*—H···*A*
N1—H*N*1···O2^i^	0.80 (3)	2.15 (4)	2.929 (4)	166
C3—H3···O3^ii^	0.93	2.60	3.463 (3)	155
C10—H10···O2^i^	0.93	2.60	3.404 (3)	145
C15—H15*B*···O3^iii^	0.96	2.36	3.323 (3)	178
C6—H6···O1	0.93	2.46	2.861 (4)	106

Symmetry codes: (i) −*x*, *y*, −*z*+1/2; (ii) *x*, *y*−1, *z*; (iii) *x*, −*y*+2, *z*+1/2.
